# Pros and cons of estimating the reproduction number from early epidemic growth rate of influenza A (H1N1) 2009

**DOI:** 10.1186/1742-4682-7-1

**Published:** 2010-01-07

**Authors:** Hiroshi Nishiura, Gerardo Chowell, Muntaser Safan, Carlos Castillo-Chavez

**Affiliations:** 1 PRESTO, Japan Science and Technology Agency, Honcho 4-1-8, Kawaguchi, Saitama, 332-0012, Japan; 2 Theoretical Epidemiology, University of Utrecht, Yalelaan 7, Utrecht, 3584CL, The Netherlands; 3 Mathematical and Computational Modeling Sciences Center, School of Human Evolution and Social Change, Arizona State University, Tempe, AZ, 85282, USA; 4 Fogarty International Center, National Institutes of Health, Bethesda, MD, 20892, USA; 5 Department of Mathematics, Faculty of Science, Mansoura University, Mansoura, 35516, Egypt; 6 Santa Fe Institute, Santa Fe, NM, 87501, USA

## Abstract

**Background:**

In many parts of the world, the exponential growth rate of infections during the initial epidemic phase has been used to make statistical inferences on the reproduction number, *R*, a summary measure of the transmission potential for the novel influenza A (H1N1) 2009. The growth rate at the initial stage of the epidemic in Japan led to estimates for *R *in the range 2.0 to 2.6, capturing the intensity of the initial outbreak among school-age children in May 2009.

**Methods:**

An updated estimate of *R *that takes into account the epidemic data from 29 May to 14 July is provided. An age-structured renewal process is employed to capture the age-dependent transmission dynamics, jointly estimating the reproduction number, the age-dependent susceptibility and the relative contribution of imported cases to secondary transmission. Pitfalls in estimating epidemic growth rates are identified and used for scrutinizing and re-assessing the results of our earlier estimate of *R*.

**Results:**

Maximum likelihood estimates of *R *using the data from 29 May to 14 July ranged from 1.21 to 1.35. The next-generation matrix, based on our age-structured model, predicts that only 17.5% of the population will experience infection by the end of the first pandemic wave. Our earlier estimate of *R *did not fully capture the population-wide epidemic in quantifying the next-generation matrix from the estimated growth rate during the initial stage of the pandemic in Japan.

**Conclusions:**

In order to quantify *R *from the growth rate of cases, it is essential that the selected model captures the underlying transmission dynamics embedded in the data. Exploring additional epidemiological information will be useful for assessing the temporal dynamics. Although the simple concept of *R *is more easily grasped by the general public than that of the next-generation matrix, the matrix incorporating detailed information (e.g., age-specificity) is essential for reducing the levels of uncertainty in predictions and for assisting public health policymaking. Model-based prediction and policymaking are best described by sharing fundamental notions of heterogeneous risks of infection and death with non-experts to avoid potential confusion and/or possible misuse of modelling results.

## Background

The reproduction number, *R*, the average number of secondary cases generated by a typical (or "average") single primary case, of influenza A (H1N1) 2009 is a summary measure of the transmission potential in the population of interest. It has been estimated using the early epidemic growth data in different locations across the world [1-12]. The estimations have been based primarily on models that include one or a limited number of aspects of heterogeneous transmission. The scientific community has been attracted to *R *because it provides a first aggregated measure of the overall transmissibility of an emerging infection [13]. Further, the estimate of *R*, based on homogeneously or nearly homogeneously mixing population models that by design ignore most individual differences, is not only used to assess the initial growth of an epidemic but also the extent to which the population will experience infection by the end of a first pandemic wave [14,15]. Except for a unique study estimating *R *using a data set of international spread [7], the exponential growth rate, *r*, of cases during the initial epidemic phase has been investigated using a simple procedure that involves translating *r *into *R *through the use of the estimator *R *= 1/*M*(-*r*) where *M*(-*r*) is the moment-generating function of the generation time distribution, given the growth rate *r *[16]. Naturally, the higher the growth rate *r *of the number of cases, the larger the estimate of *R*.

The majority of *R *estimates for this ongoing pandemic have ranged from 1.1-1.8 [17] while our estimate of *R *in Japan was in the range of 2.0-2.6 under the assumption of a mean generation time of 1.9 days through May 2009 [5]. The most plausible reason for this estimate, as noted in our earlier study [5], involved the role of initial conditions as the very early growth was driven by the high contact rates that are common to school settings [17,18]. In addition to the phenomenological explanation, it is important to assess whether or not the methodology used to estimate *R *was adequate. We do this here using data that go beyond those used in our estimation of *R *for the earlier epidemic period of May 2009 in Japan. Here we provide an updated estimate of *R *for the novel influenza A (H1N1) 2009 in Japan, summarising the relevant methodological issues in estimating *R *from the growth rate of cases and initiating a dialogue on how estimates of the transmission potential should be shared with non-experts, including the general public.

## Discussion

### The epidemic data in Japan

**Figure 1:**
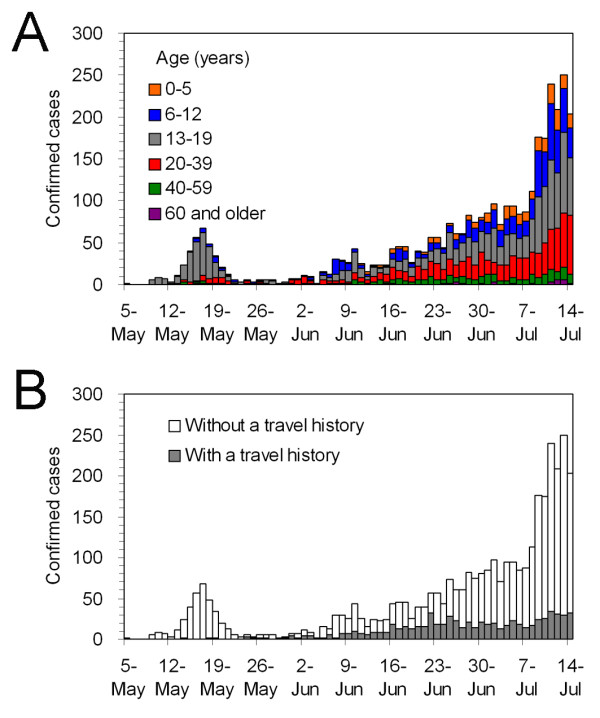
**Temporal distribution of confirmed cases of influenza A (H1N1) 2009 virus infection in Japan from May to July 2009 (n = 3,480)**. All the confirmed cases were diagnosed by RT-PCR. The horizontal axis represents the date of onset. Cases are stratified by (A) age and (B) travel history. Here "cases with travel history" are associated with overseas travel within 10 days preceding onset of illness and those with such a history are referred to as imported cases in our analysis.

Figure 1 shows the epidemic curve of influenza A (H1N1) 2009 for Japan from May to July 2009. Starting with the illness onset of an index case on 5 May, 4986 confirmed cases, all diagnosed by means of RT-PCR, were reported to the government during this period. On 22 July, the Ministry of Health, Labour and Welfare of Japan decided not to mandate its local health sectors to notify all the confirmed cases, and thereafter the local sectors gradually ceased counting all the cases. The first pandemic wave in Japan continued to grow steadily thereafter hitting the first peak in November [19].

Since our original data indicate that the 97.5 percentile point of the reporting delay distribution (i.e., the time from illness onset to notification) is 8 days, we analyse a total of 3480 cases that developed the disease on or before 14 July. Figure 1A shows the temporal distribution stratified by age-group of all identified cases. Of the 3480 cases, 67.0% were among individuals 19 years of age or less. The population of those aged from 20-39 years accounted for 24.2% of the total, and the remaining (older adult) cases accounted for only 8.8%. The contributions from imported cases to the early epidemic growth in this island nation, in addition to the local (indigenous) transmissions, are also critical (Figure 1B). Of the 3840 confirmed cases, 694 (19.9%) had a history of overseas travel within 10 days preceding the onset of illness, and we refer to them as imported cases in the present study.

### Growth rates of two different phases

**Figure 2:**
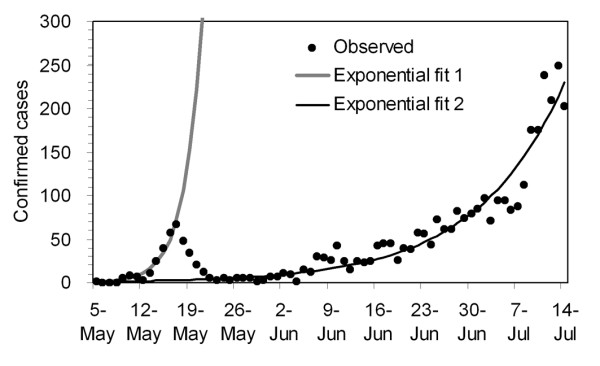
**Simple extrapolation of the exponential growth of cases**. Two exponential fits are compared with the observed number of confirmed cases. Exponential fit 1 employs the data set from 5 May to 17 May during which clusters of cases in a few high schools fuelled the epidemic. Exponential fit 2 draws the best fit to the data from 29 May to 14 July representing the spread of influenza into the wider population. The growth rates for fits 1 and 2 are estimated at 0.37 and 0.08 per day, respectively.

We proceed to compare two different growth rates (Figure 2) in order to explore the patterns that led to our past *R *estimates for Japan in [5]. The growth rates of cases in the very initial phase (i.e., from 5 to 17 May), which corresponds to the period examined in our earlier study [5], and those that followed the generation of secondary cases caused by school clusters (i.e., from 29 May to 14 July) are compared. Over these periods we observe that the proportion of cases attributed to the 0-19 age grouping decreased from 83.0% to 67.0%.

We model the expected value of the incidence of illness onset at calendar time *t *as E(*c*(*t*)) = *k*exp(*r*(*t*-*τ*)) where *k *is a constant, *r *is the growth rate of the corresponding period, and *τ *is the starting time point of exponential growth (assumed as 5 May and 29 May, respectively). Minimizing the sum of squares between the observed data and expectation, *r *is estimated as 0.37 and 0.08 per day, respectively, for the former and latter periods. The estimate for the former period is smaller than that reported in our earlier study in May (i.e., 0.47 per day) [5], because of our use of refined dates of onset and the use of a simpler statistical method in the present study. The estimates of the exponential growth rates differ by almost a factor of five (i.e., 0.37/0.08) in the two windows in time, indicating that the cases in the former period experienced a 1.3 times greater daily growth rate (i.e., exp(0.37)/exp(0.08)) than those in the latter period. A glance at the age-specific data show that the disease spread from an initial cluster that mix primarily in an assortative manner into the "general" Japanese population is the most likely key to this dramatic difference. Since the latter period reflects the early population-wide spread of H1N1 involving the entire Japanese community, *R *for this period is estimated using the following methodology.

### Modelling methods

We employ an age-structured model to derive an estimate for *R *since the transmission of influenza A (H1N1) 2009 is known to differ greatly among age groups [1,3,5,9]. Spatial heterogeneity, social heterogeneity (e.g. differing patterns of transmission between household-, school- and workplace-settings), or potential changes in behaviour are mostly ignored.

The square matrix with generic entry *R*_ij_, the average number of secondary cases in age-group *i *generated by a single primary case in age-group *j*, is referred to as the next-generation matrix [20]. The reproduction number *R *is defined as the dominant eigenvalue of the next-generation matrix [21]. Since the observed data come as daily reports, we consider the incidence of indigenous, *c*_i, t_, and imported cases, *b*_i, t_, of age-group *i *developing the disease on day *t *in discrete time. Using *R*_ij_, the multi-type renewal process, yielding the conditional expectation of indigenous cases on day *t*, is written as(1)

where *α *is the relative contribution of imported cases to secondary transmission as compared to indigenous cases (0 ≤ *α *≤ 1) and *g*_s _is the discretized density function of the generation time of length *s *days. We introduce the relative reduction *α *because the physical movements of those with a history of overseas travel were partly restricted during the early epidemic phase in Japan, reducing the number of secondary transmissions. Also, the imported cases most often developed the disease shortly before or after entering Japan. The density of the generation time, *g*_s_, is calculated as follows:(2)

where *G*(*s*) is the cumulative distribution function of the generation time distribution, which we assume to be known and to follow a gamma distribution. In the early modelling studies, the mean generation time was estimated at 1.9 days [1], 2.6-3.2 days [3] and 2.5 days [4]. From contact tracing data in the Netherlands, the mean and standard deviation (SD) were estimated at 2.7 and 1.1 days, respectively [22]. We adopt 2.7 days as the mean and fix the coefficient of variation to 40.7% as calculated from the Dutch study. We partly address issues of uncertainty by measuring the sensitivity of *R *to differing mean generation times ranging from 2.1 to 3.3 days.

*R*_ij _is modelled as(3)

where *R *is the reproduction number to be estimated (i.e., scalar quantity), *s*_i _measures the susceptibility of age-group *i *given a contact, and *m*_ij _is the frequency of contact made by an individual in age-group *j *with that in *i *(which is assumed known and is extracted from a contact survey in the Netherlands [23]); let *S *and *M *be square matrices. *S *is the diagonal matrix in which the diagonal elements (*i*, *i*) are *s*_i _and the entries outside the main diagonal are all zero. The (*i*, *j*) element of *M *is *m*_ij _with which we adopt frequency-dependent assumption, and we ignore more detailed contact including the "type" and "duration" [24]. We normalize the product *SM *(i.e., the dominant eigenvalue of *SM *is set to 1) so that *R *scales the next-generation matrix. We aggregate the population into six discrete age groups (0-5, 6-12, 13-19, 20-39, 40-59 years and 60 and older) in order to be able to adhere to the precision of the contact survey [23]; consequently, the next-generation matrix has dimensions 6 × 6 (36 elements).

We estimate eight parameters (i.e., *R*, *α *and *s*_i _for six age-groups) using the renewal equation (1). We assume that variations in secondary transmissions are appropriately captured by a Poisson distribution [25]. The conditional likelihood of observing *c*_i, t _on day *t *given the series of foregoing indigenous cases *c*_j, 0_, *c*_j, 1_, ..., *c*_j, t-1 _and of imported cases *b*_j, 0_, *b*_j, 1_, ..., *b*_j, t-1_, respectively, for all age-groups *j*, is given by(4)

where E(*c*_i, t_) is the conditional expectation (i.e., the right-hand side of (1)) and *c*_i, t _is the observed number of cases of age-group *i *on day *t*. Maximum likelihood estimates of the parameters are obtained by minimizing the negative logarithm of (4) with the 95% confidence intervals (CI) derived from profile likelihood.

### Modelling results

**Figure 3:**
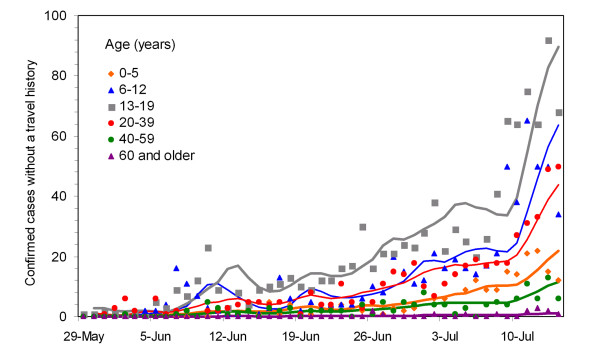
**Model prediction**. Observed (dots) and predicted (lines) age-specific numbers of confirmed cases as a function of onset time are compared. The prediction on day *t *was conditioned on observations from days 0 to (*t*-1).

Figure 3 compares the observed and predicted numbers of confirmed cases. The conditional expectation approximately captures the observed age-specific patterns of incidence. The maximum likelihood estimate of the next-generation matrix, *R*_ij_, is(5)

Those aged from 13-19 years appear to be able to maintain the transmission by themselves (i.e., meeting the definition of maintenance host, *R*_33 _> 1 [26]). Nevertheless, age groups 1 and 2, children aged from 0-12 years, appear incapable of maintaining transmission (i.e., the dominant eigenvalue of the 2 × 2 matrix involving transmissions among and between those aged from 0-5 and 6-12 years is less than 1). The maximum likelihood estimate of *R *is 1.28 (95% CI: 1.23, 1.33). The relative contribution of imported cases to secondary transmission, *α*, is estimated at 0.15 (standard error = 0.14).

**Figure 4:**
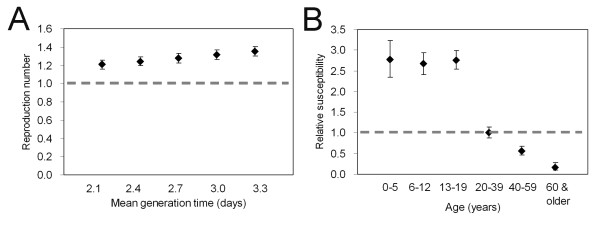
**Parameter estimates and sensitivity analysis**. Panel A examines the sensitivity of the reproduction number to different mean lengths of the generation time ranging from 2.1 to 3.3 days. Panel B shows the estimate of the age-specific relative susceptibility. The expected value of susceptibility for those aged 20-39 years was taken as the reference. In both panels, the whiskers extend to the upper and lower 95% confidence intervals based on the profile likelihood.

Figure 4A examines the sensitivity of *R *to different mean generation times. If we adopt 2.1 days as the mean, *R *is estimated at 1.21 (95% CI: 1.16, 1.26). If we adopt 3.3 days, *R *is 1.35 (95% CI: 1.30, 1.41). Figure 4B captures relative susceptibilities, using those aged from 20-39 years to define the susceptibility baseline. The age-groups 0-5, 6-12 and 13-19 years appear to be 2.77 (95% CI: 2.35, 3.24), 2.67 (95% CI: 2.41, 2.95) and 2.76 (95% CI: 2.55, 2.98) times more susceptible than adults aged 20-39 years. On the other hand, those aged from 40-59 years and 60 years and older are 0.56 (95% CI: 0.45, 0.68) and 0.17 (95% CI: 0.09, 0.28) times as susceptible than those aged 20-39 years. It should be noted that the qualitative pattern of age-dependent susceptibility agrees well with the results of immunological studies [27,28] and a hypothesis about its underlying mechanisms [29].

### Limitation of the growth rate *r*

As expected from the greatly differing exponential growth rates between early May and from 29 May to 14 July (Figure 2), the reproduction number for the latter period, ranging from 1.21 to 1.35, is much smaller than our previously reported estimate for the former time period when the transmission was mainly confined to school settings [5]. The estimate in the latter period is consistent with the estimates of *R *in other countries [14,16]. The situation is not straightforward, however, as the estimation was carried out using confirmed cases (which may be biased towards severe cases). Further, it should be noted that various interventions, including reactive school closure and contact tracing, were instituted during the whole period of observation, so the *R *value for the latter period and especially the entries *R*_ij _involving school children might potentially lead to underestimates for *R *in the present study.

Since the small outbreak in the former time period was restricted to a limited number of schools and the contacts made by the students in Osaka and Kobe (and as Japan was unique in successfully "containing" the local school-based outbreak before actual pandemic overshoot), the depletion of susceptibles in May and undiagnosed cases are unlikely to have played a significant role in our estimates of a smaller *R *for the epidemic in the latter transmission period, which saw the pandemic takeover. Rather, as we discussed above, the local networks of interactions (i.e., transmission within networks that connect to other networks in time), and consequently the initial conditions (i.e., which network gets infected first), played a key role in our estimates for the initial outbreak growth. The earlier estimates of *R *captured the initial role in the generation of secondary cases from schools where the frequency of transmission among school children greatly exceeded those of the community (and the "type" of their contact is perhaps more dense (or close) than those in the community [24,30]). Although the sensitivity of *R *to differing mean generation times was examined within a relatively narrow range, this aspect could not account for the high *R *estimates obtained in Japan [5].

One important conclusion is drawn from the present study. The lessons learnt from our estimation over the two windows in time has forced us to revisit the role of using summary statistics to characterize transmission potential from the data generated by heterogeneous contact patterns. As a network expands, the structure of networks involved in transmission changes, and consequently the summary statistics of cases, also change in time [13]. The initial summary statistics therefore depend in a rather critical way on the initial conditions (i.e., where and how the disease was introduced) [31], which is not always captured well by homogeneous mixing models. Since the very early stage of this epidemic alone involves primarily a few specific sub-groups of the population, it is difficult to quantify the next-generation matrix fully and estimate a reproduction number that adequately captures the transmission potential for the entire population. Whereas the next-generation matrix includes representative levels of population heterogeneity, the infected individuals during the very initial epidemic stage were clearly not representative of the entire population of interest. The use of the next-generation matrix involves the introduction of a "typical" infectious individual into the population, but such an individual cannot be properly characterized if the matrix involves unavoidable approximations (due to limited availability of structured data) when an outbreak happens to be mostly confined to a single cluster whose average individual is "atypical" of the entire population.

The previously reported expected value, *R *= 2.3, for the May outbreak might well approximate the intensity of transmissions in schools (and indeed, is consistent with the estimate in school settings in the USA [3]). This is another example of what is often referred as core group effects in the epidemiological literature [32]. Naturally, the use of the empirical data from school clusters does not provide sufficient information to carry out precise estimation of the age-dependent next-generation matrix, so the resulting dominant eigenvalue in the earlier study should not be regarded as *R *but rather as a measure of transmissibility conditioned on the initial conditions. The need for the collection of additional data may be critical when age-specific transmission is highly assortative and/or when age-specific susceptibility is highly heterogeneous (as is the case for influenza A (H1N1) 2009). Summary statistics based on highly aggregated populations are in general not helpful in identifying the pressure points of a heterogeneous network, which is essential in the identification and assessment of the most effective (e.g., age-specific) intervention policies. In other words, the finer details of epidemic data (i.e., epidemiological information at a local level, e.g., active surveillance of cases) need to be taken into account in the modelling. Not only school outbreaks, but also other social factors and settings (e.g. transportation, hospital settings and mass gatherings) can play enhancing or reducing transmission roles.

In addition to the challenges posed by our need to average over different levels of heterogeneous mixing, quantification of the growth rates involves the additional challenges that come from underreporting, notably ascertainment of cases and reporting bias. Further, imputation of onset dates for missing data is sometimes required, and moreover, the time-varying reporting frequency may even call for naïve adjustment of the growth rate of confirmed cases by the growth rate of hospitalized (or other severe portions of) cases [4]. The data set we examined in Japan involved contact tracing efforts at all local levels, so the growth rate of confirmed symptomatic cases is thought to have captured the actual increase in infection appropriately. Nevertheless, achieving precise estimation of incidence for this mild disease remains an open question, particularly if the proportion of asymptomatic infections among the total of infected individuals is high.

### Is the epidemic growth rate useless?

Despite our earlier suggestion of the "biased" estimate of the next-generation matrix, it should be noted that we do not argue that the early growth rate is no longer be used but rather that the context of its use when appropriate should be clarified. The growth rate of cases is, as with most inferences from statistical modelling, context dependent (e.g., presence of initially infected cluster). Given the precise estimate of the generation time distribution, the exponential growth rate is appropriately translated to the reproduction number for a single population [16]. This is also the case, for example, for the multi-type epidemic as outlined below. Discarding imported cases, the continuous-time version of our renewal process (1) is written as(6)

Equation (6) assumes that the generation time is shared among sub-populations. If we further assume that the intrinsic growth rate *r *is identical among sub-populations then the incidence *c*_i_(*t*) can be written as ([33]):(7)

where *k *is constant and *ω*_i _is the leading eigenvector of the next-generation matrix. Replacing the right-hand side of (7) in (6) leads to(8)

That is,(9)

and thus the estimator of *R *is given by 1/*M*(-*r*) (see Background) [16]. Hence, and not surprisingly, as long as the intrinsic growth rate and generation time are the same among sub-populations, the estimator of *R *for the multi-type epidemic model can be identical to that of single-host epidemic model [34].

The incorporation of additional levels of detail into the basic model used to generate growth estimates depends on the model's ability to capture the underlying transmission dynamics in the data and the purposes of the research questions or public health policymaking goals. These issues are particularly relevant when a clustering of cases is observed [35,36]; as we saw in Japan, clusters of cases caused a delay in accurately estimating the true population average of transmissibility. The epidemic growth rate remains a useful quantity for estimating the transmission potential at the population level in the absence of obvious clusters of cases and as long as the approximately modelled transmission sufficiently captures the actual heterogeneity. We start from the premise that the use of heterogeneous mixing models is essential in the assessment of critical theoretical claims and policymaking decisions. Hence, it is worth noticing that in this context, technical questions remain regarding the use and applicability of the exponential growth rate. They include (i) the development of methods for estimating the generation time distribution and (ii) the determination of an appropriate length of the exponential growth period [37-39].

### How should we communicate the estimate?

Without doubt *R *is the most widely used measurement of transmissibility and there are many good reasons why this is so. It has a simple formula and it is the simplest and most interpretable quantity to communicate to non-experts. Its limitations become evident when specific decisions must be made including, for example, who should be vaccinated first. Precise estimates of the next-generation matrix capture detailed epidemic dynamics that are key to answering questions like the one posed, but its estimation requires age- and risk-group structured data and a clear identification of the correct exponential growth time window. In the context of the pandemic from 2009, gathering age-specific transmission dynamics information is of the utmost relevance to prediction and policymaking. For instance, given that *R *= 1.28, one may predict the final size of epidemic, *z*, the proportion of those who will experience infection by the end of epidemic, by using the final size equation (based on a homogeneous mixing model),(10)

Iteratively solving (10), *z *is estimated to be 40.3%. Similarly, *R *= 2.3 for a homogeneously mixing population is translated to z = 86.2%. Nevertheless, if we have the next-generation matrix, *R*_ij_, the final size *z*_i _of host *i *is written as ([40])(11)

Using our estimate in (5), the corresponding *z*_1_-*z*_6 _are estimated at 16.2, 37.1, 47.7, 29.0, 8.7 and 1.0%, respectively. Using the age-specific population size *N*_i_, the final size *z *for the entire population is calculated as the weighted average,(12)

Extracting the age-specific population estimate in Japan [41], *z *is estimated at 17.5%, not surprisingly much less than predicted by (10), a value that is indeed close to the actual range of the impact of first pandemic wave in Northern Hemisphere countries [17]. The "real" value of *z *may be even smaller if we account for additional levels of heterogeneity in transmission. The reproduction number, *R*, for the entire population may be useful for obtaining a rough estimate of how much vaccine we need (e.g., deciding the total number of vaccines to be manufactured), while *R*_ij _is far more essential for structuring the most effective strategy of vaccination and planning the optimal prioritization schemes [42,43]. Given that *R *can also be calculated from *R*_ij_, communicating *R*_ij _rather than *R *to the general public would be the most informative strategy of science communication for modelling results. When one explains the concept of *R*_ij _to non-experts, it's ideal to mention the limitation due to its nature of approximation because of limitations in structured data in any empirical observation.

The case fatality ratio (CFR), an epidemiological measurement of virulence, would also benefit from the use of detailed (e.g., age-structured) information. Whereas the confirmed CFR (cCFR) for the entire population conditioned on confirmed cases has been estimated at approximately 0.5% during the very early stage of the pandemic [1,44,45], the symptomatic CFR (sCFR), which is conditioned on symptomatic cases, later appeared to be 0.048% [46]. The CFR estimate for the entire population is regarded as a summary measure of virulence, so the reduced order of virulence (approximately by a factor of 10) provides not only more accurate information but a more useful measure for assessing the impact of this pandemic. Nevertheless, knowledge based on additional information would be even more useful. Ideally, we would like to compare epidemiologically the virulence between different settings (e.g., comparison between age groups or countries) and to use this knowledge in the development of relevant public health policy. Understanding the heterogeneous risks of death by age- and risk-groups is critical in the effective design of intervention strategies. In other words, apart from discussing the changing assessment of the CFR as a whole, CFR_i _for age-group *i *is probably more informative than CFR for the entire population.

## Conclusions

We analysed the temporal distribution of the influenza A (H1N1) 2009 epidemic in Japan from May-July 2009, estimating the reproduction number for the time period from 29 May to 14 July -- a follow-up to our earlier estimates derived primarily from data on school outbreaks in May. The updated estimate of *R*, an average over a wider spectrum of the Japanese population, ranged from 1.21 to 1.35. Our higher estimate reported in [5] was based on a data set that was incapable of quantifying the next-generation matrix for the whole country from the growth rate of cases in a restricted school setting alone. The upshot of this analysis is tied to the fact that in regions where disease transmission is highly heterogeneous the initial conditions will have a strong effect on the variation in estimates of the rate of epidemic growth. To quantify *R *and *R*_ij _appropriately from the growth rate of cases, it is essential that the model not only captures the underlying transmission dynamics behind the empirical data but also that we have sufficiently structured data for each sub-population to estimate the role of variability in our estimates. In addition to extending the window in time for developing accurate population level measurements [39], it may be useful to examine additional epidemiological information (e.g., active surveillance of local transmission patterns and the severe portion of cases including hospital admissions).

Communicating *R *to non-experts is simpler than *R*_ij_, but *R*_ij _contains the information that is most useful for the development of specific intervention efforts. Developing simple interpretations and effective ways of communicating *R*_ij _(i.e., the average number of secondary cases in host *i *generated by a single primary case in host *j*) must be pursued. This retrospective study highlights the importance of incorporating the concept of heterogeneous risks of infection and death in the context of the assessment of pandemic potential.

## List of abbreviations

*R*: the reproduction number; RT-PCR: Reverse transcription polymerase chain reaction; CI: confidence intervals; SD: standard deviation; CFR: case fatality ratio.

## Competing interests

The authors declare that they have no competing interests.

## Authors' contributions

HN conceived of the study. HN developed methodological ideas, implemented statistical analyses and drafted the manuscript. GC, MS and CCC revised the model and helped improve the manuscript. All authors read and approved the final manuscript.
